# Unlocking the Power of the Mediterranean Diet: Two in One—Dual Benefits for Rheumatic and Thyroid Autoimmune Diseases

**DOI:** 10.3390/nu17081383

**Published:** 2025-04-19

**Authors:** Maria Cristina Barbalace, Rossella Talotta, Federica Rapisarda, Valeria D’Amico, Martina Laganà, Marco Malaguti, Alfredo Campennì, Salvatore Cannavò, Silvana Hrelia, Rosaria Maddalena Ruggeri

**Affiliations:** 1 Department for Life Quality Studies, Alma Mater Studiorum, University of Bologna, 47921 Rimini, Italy; marco.malaguti@unibo.it (M.M.); silvana.hrelia@unibo.it (S.H.); 2 Rheumatology Unit, Department of Clinical and Experimental Medicine, University Hospital “G. Martino”, 98124 Messina, Italy; rtalotta@unime.it (R.T.); feduccia.rapisarda@gmail.com (F.R.); damico.valeria95@libero.it (V.D.); 3 Endocrinology Unit, Department of Human Pathology of Adulthood and Childhood DETEV “G. Barresi”, University of Messina, 98125 Messina, Italy; martinalagana5@gmal.com (M.L.); cannavos@unime.it (S.C.); rmruggeri@unime.it (R.M.R.); 4 Unit of Nuclear Medicine, Department of Biomedical and Dental Sciences and Morpho-Functional Imaging, University of Messina, 98125 Messina, Italy; acampenni@unime.it

**Keywords:** Mediterranean diet, polyphenols, polyunsaturated fatty acids (PUFAs), rheumatoid arthritis, spondyloarthritis, systemic lupus erythematosus, Sjogren’s syndrome, autoimmune thyroiditis, oxidative stress

## Abstract

In recent years, autoimmune diseases are becoming more and more prevalent worldwide, with this rapid rise being influenced by environmental factors linked to lifestyle changes in modern societies. In this context, the role of diet has been the topic of extensive research as evidence has mounted that particular dietary patterns may contribute to or modulate autoimmunity. The present review specifically focuses on the Mediterranean diet (MD) as a whole dietary pattern, and on its peculiar components, such as n-3 polyunsaturated fatty acids (PUFAs), polyphenols and fiber. We explored their potential benefits in a spectrum of both systemic and organ-specific autoimmune disorders, including rheumatic diseases (like rheumatic arthritis and systemic lupus erythematosus), and thyroid diseases (like Hashimoto’s thyroiditis), since they often occur in the same individuals. Here, we offer a comprehensive review about the influence of dietary factors on these autoimmune diseases and potential translation into therapeutic interventions, as an adjuvant therapeutic approach to improve autoimmunity-related outcomes.

## 1. Introduction

Autoimmune diseases encompass a broad spectrum of systemic or organ-specific diseases characterized by the abnormal development of an immune response against self-antigens. Autoimmune diseases have complex pathogenesis that depends partly on a genetic predisposition and partly on environmental factors. Next-generation sequencing studies have shown that autoimmunity is associated with hundreds of genomic variants [1]. The genes involved are usually of crucial importance for maintaining a functional immune response against pathogens and preventing autoreactivity. Environmental triggers include chemical, biological, and physical factors that can activate the immune response by various mechanisms, such as molecular mimicry, post-translational modification of proteins, interferon type I production, epitope spreading, or superantigen presentation [2,3,4]. T and B lymphocytes are the main players in the immunological scenario of autoimmune diseases, which react directly or indirectly (by synthesizing autoantibodies) against self-antigens. However, immune cells belonging to innate immunity and non-immune cells such as epithelial, endothelial cells, and fibroblasts also significantly contribute to inflammation and final tissue damage by producing pro-inflammatory cytokines and other mediators. In addition, regulatory T cells (Treg cells), which play a central role in self-tolerance, are reduced in most autoimmune diseases [4]. The target antigens of the autoreactive T and B cells can be localized in a specific organ or distributed throughout the body. Organ-specific autoimmune diseases include Hashimoto’s thyroiditis (HT), type 1 diabetes mellitus (T1D), and Graves’ disease (GD), to mention a few. Rheumatoid arthritis (RA), seronegative spondyloarthritis (SpA), and autoimmune connective tissue diseases (CTDs), on the other hand, are characterized by systemic inflammation due to the recognition of multiple antigens in different tissues and organs. The treatment of autoimmune diseases is usually based on the use of immunosuppressive drugs or substitution therapy for hormonal dysfunction. Recently, international guidelines have begun to include hygienic–dietetic recommendations in the therapeutic management of such patients [5,6,7,8]. Dietary habits and the intake of antibiotics can affect the gut microbiota and trigger the activation of immunological pathways that can culminate in autoimmunity. In fact, the gastrointestinal tract can harbor up to 70% of the total lymphocyte population, whose phenotype can be modulated by the composition of commensal bacteria [5]. Several studies confirm that dysbiosis occurs in a variety of autoimmune diseases, including RA, HT, and T1D, to mention a few [8,9,10]. In addition, dysfunction of tight junctions is a common feature of autoimmune diseases. It leads to altered intestinal permeability, which is associated with increased transport of antigens from the intestinal lumen and the risk of malabsorption of macro- or micronutrients such as vitamin D [11].

A diet high in fat, salt, and sugar can promote T helper 17 (Th17) differentiation to the detriment of Treg cells. Conversely, a high fiber intake may promote the proliferation of *Faecalibacterium prausnitzii* species, which could prevent inflammation [5]. Moreover, the consumption of large amounts of fats, animal proteins, and refined sugar favors a condition of oxidative stress with reactive oxygen species (ROS) overproduction, while a low intake of fruits and vegetables causes a lack of exogenous antioxidants [12,13]. Noteworthy, thermally processed foods, particularly those rich in lipids and proteins, represent a plentiful source of exogenous advanced glycation end products (AGEs), that act synergistically to the endogenous formation, increasing AGEs load and related glycative/oxidative stress, whereas plant-derived foods are rich in polyphenols, that act as antioxidant and antiglycation agents [14,15]. Recently, our group provided evidence that high dietary intake of animal foods (namely, meat and fats) is associated with higher levels of the oxidants AGEs and lower levels of antioxidants [13].

Scientific evidence also shows that moderate-to-high alcohol consumption is associated with RA flares, whereas low–moderate alcohol consumption seems to be protective against autoimmune hypothyroidism and Graves’ disease [16,17,18].

Most micro- and macronutrients that have been shown to play a beneficial role in inflammation are included in the Mediterranean diet (MD). The MD consists of a diet rich in fiber content, vegetable food, olive oils, and a moderate intake of fish, poultry, and wine. Contrary to the Western diet, the MD contains a minimal quote of processed food, refined sugar, or red meats. The knowledge about the immunomodulatory influence of the diet has been increased and the role of whole dietary patterns like the MD has emerged in recent years. Indeed, the MD could potentially modulate inflammatory pathways for its antioxidant and anti-inflammatory properties [19]. Part of these effects may be attributed to its role in shaping intestinal microbiota by favoring microbial diversity and the growth of beneficial bacteria, such as *Bifidobacterium*, *Lactobacillus*, and *Faecalibacterium prausnitzii*, which are known for their anti-inflammatory effects [20]. For these reasons, the MD should represent a healthy dietary model in the setting of autoimmune disorders, since it could be defined as the model opposed to the Western diet, and it can be recommended to patients with autoimmune diseases [21].

Data from the literature, for instance, support the beneficial effects of the MD on RA [22,23,24,25,26,27], spondiloartrhitis [28,29], CTD [30,31,32], as well as autoimmune thyroiditis [11,13,21]. Also, several components of plant-derived products may be responsible for the beneficial effects of the MD against inflammation, oxidative stress, and autoimmunity, as detailed below.

The aim of this review is to provide relevant evidence for the beneficial effects of the MD in a range of systemic or organ-specific autoimmune diseases, with particular emphasis on endocrine and rheumatic disorders that may occur in the same individuals due to common pathogenic mechanisms.

## 2. Materials and Methods

An extensive literature search was carried out independently on online databases (MEDLINE via PubMed, ISI Web of Science, and Scopus). The following keywords and MESH terms were used: “Mediterranean diet” AND “rheumatoid arthritis” OR “spondyloarthritis” OR “systemic lupus erythematosus” OR “Sjögren’s syndrome” OR “systemic sclerosis” OR “Hashimoto’s thyroiditis” OR “autoimmune thyroiditis” OR “Graves’ disease”. Using the same selected diseases, we also searched the literature for associations with individual MD components (dietary fiber, ω-3 PUFAs, polyphenols, resveratrol, vitamins and micronutrients). A 2-step selection process was conducted by 3 reviewers independently of each other. Articles pertinent to the aim of the review, including reviews, metanalyses and original studies, were selected on the basis of the relevance of the title and abstract in the topic by three reviewers (M.L., F.R., V.D.), and then were critically evaluated by three other reviewers (R.M.R., M.C.B., L.C.). Articles written in the English language and published in peer-reviewed journals were included in the present review and discussed in the sections below.

## 3. Beneficial Effects of the Components of the Mediterranean Diet in Autoimmune Diseases

The term “Mediterranean diet” was coined in 1960 by Ancel Keys during his pioneering study called “Seven Country Study” where, for the first time, he demonstrated that the habits and traditions regarding the foods of the populations living in the Mediterranean basin could impact their health and in particular were associated with lower mortality for coronary heart disease [33]. In 2010, UNESCO included the MD in the Intangible Cultural Heritage List because it represents “a set of skills, knowledge, practices and traditions ranging from the landscape to the table, including the cultivation, harvesting, fishing, preservation, processing, preparation and, in particular, consumption of food characterized by a nutritional pattern that has remained constant over time and space, always respecting the beliefs of each community” [34].

Over time, the beneficial effects derived from the MD were expanded over the first observations of Keys including an inverse correlation with the incidence of neurodegenerative diseases [35,36], metabolic syndrome [37,38], cancer [39,40], obesity and type 2 diabetes mellitus [41,42].

The MD includes a high intake of plant-based foods like grains, seasonally available fruits and vegetables, legumes, nuts, seeds and olives, alongside fish and seafood, simultaneously with a moderate amount of cheese, dairy and meat. Extra virgin olive oil is the primary fat source, known for its high nutritional quality due to its rich content of bioactive compounds, mainly phenols. A separate discussion should be conducted about wine, especially red wine, that is consumed in moderation, and often during meals. The positive effects of wine consumption are currently a topic of debate, particularly in both the scientific community and beyond [43]. However, it is important to emphasize the benefits of moderate wine consumption during meals, as typically practiced in the MD. We must underline that the MD is not just a nutritional model; it also embodies social interaction, love for food and a deep connection to the land and its traditions. Indeed, our aim is not to present alcohol as a health-promoting substance or to encourage its consumption for potential benefits. Instead, we seek to highlight the role of moderate alcohol intake—especially red wine—within the context of the MD, and to clarify that there is no conclusive evidence supporting total abstinence to lower the risk of developing autoimmune diseases.

**Figure 1:**
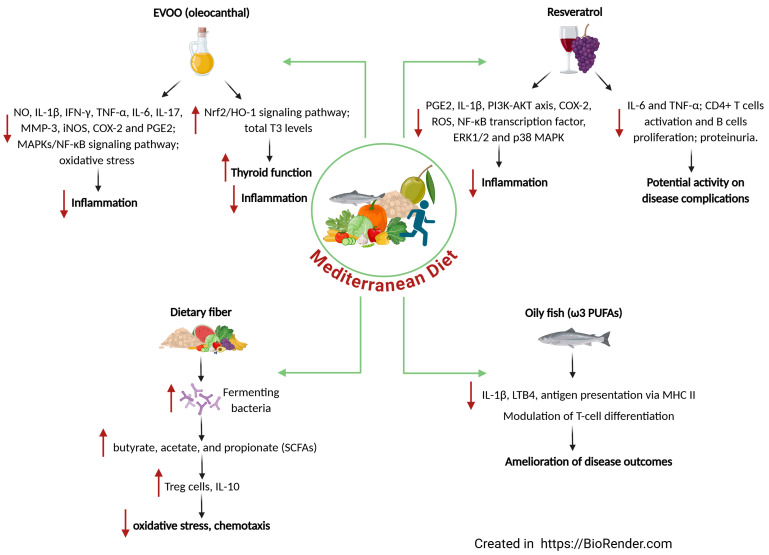
The key components of the Mediterranean diet and their possible role in the prevention/treatment of autoimmune diseases. ↓ Decreased; ↑ Increased.

The components of the MD have been studied for their anti-inflammatory effects. For example, the intake of small oily fishes (the so-called “pesce Azzurro”, rich in ω-3 PUFAs) as the main source of animal protein, and the great variability in fruit and vegetables (rich in different classes of bioactive compounds) consumed daily have already been mentioned in other studies for their great potential in counteracting the mechanisms beyond autoimmune pathologies [13,21,44]. Indeed, the sum of the individual effects of each component of MD can attenuate or even eliminate the inflammatory process that occurs in autoimmunity, making this dietary habit a strategic choice for the prevention and treatment of autoimmune diseases and their complications (Figure 1).

### 3.1. Dietary Fibers

The term dietary fiber defines those carbohydrates that are not digested in the upper gastrointestinal system nor absorbed in the small intestine [20]. Dietary fibers are, therefore, processed by intestinal microbiota through bacterial fermentation. Food with a high content of dietary fiber includes cereals, fruits and vegetables. It has been observed that the intake of fibers in daily diet increases the number of fermenting bacteria, which produce short-chain fatty acids (SCFAs), mostly represented by butyrate, acetate and propionate. SCFAs are particularly known for their anti-inflammatory properties. Indeed, they can promote the integrity of the gut barrier by enhancing the number of tight junctions, thus reducing the passage of bacterial constituents (PAMPs) in the lamina propria stratum where they could sensitize immune cells. Moreover, SCFAs can reduce the oxidative stress and chemotaxis of immune cells while increasing the number of Treg cells and the release of interleukin 10 (IL-10) [45]. It is widely recognized that the gut microbiota play a crucial role in regulating the host’s immune system through epigenetic processes. Disruptions in microbiota, known as dysbiosis, can disturb the immune balance and contribute to the onset of autoimmune diseases [46]. While the precise connection between these disruptions, dietary habits and autoimmunity remains incompletely understood, it is plausible to suggest that a beneficial microbiota composition promoted by the MD could offer protection against the development of thyroid autoimmunity [47]. Furthermore, a recent systematic review of 22 case–control studies on 915 primary Sjogren’s syndrome (pSS) patients and 2103 healthy controls (HCs) found a reduced expression of SCFA-producing bacteria in patients versus controls, thus suggesting that, also in this case, an alteration in the microbiota composition could favor the development of an autoimmune disorder [48]. Differently, in a prospective observational, non-randomized, clinical trial of 50 RA patients subjected to the MD, the authors did not find a significant correlation between SCFAs and disease activity and no differences in butyrate or propionate profile in patients undergoing MD or 7-day fasting intervention (acetate was increased in fasting) [49].

### 3.2. PUFAs

Linoleic acid (LA) and alfa-linolenic acid (α-LA) represent the precursors of ω-6 and ω-3 PUFA families, and because the human body is not able to synthesize them, they are classified as essential fatty acids, and their intake totally depends on the diet. The main difference between the two classes is the position of the double bonds on the carbon chain, starting from the methyl end of the carbon chain (the ω-carbon). ω-6 PUFAs have the first double bond at the sixth carbon, while ω-3 PUFAs have it at the third carbon. ω-6 PUFAs typically originate from animal sources. Meanwhile, oily fish, vegetable oils, green plant tissue and nuts are rich in ω-3 PUFAs. Both ω-6 and ω-3 PUFAs are essential components of phospholipid membranes and precursors of inflammatory mediators, but a balanced ω-6/ω-3 ratio is important to support the anti-inflammatory profile of ω-3 PUFAs.

Several studies evaluated the ω-3 PUFAs supplementation as a clinical intervention in controlling RA, demonstrating beneficial effects. In almost all the studies, patients enrolled who received the ω-3 PUFAs supplementation were able to reduce the consumption of NSAIDs and analgesics, demonstrating their potential as adjuvant therapy [50,51,52,53]. Clinical improvements, like improvement in the number of tender joints, were correlated with the ω-6/ω-3 fatty acids ratio from the plasma samples of the fish-oil-receiving group [54]. In a small nutrition study involving predominantly patients with active RA, the recommendation to consume seafood correlates with better disease outcomes [55]. A recent meta-analysis analyzing 23 similar clinical studies revealed consistent findings on joint swelling, pain and morning stiffness [56].

Regarding the mechanisms behind positive effects, there can be several. ω-3 PUFAs can suppress the levels of inflammatory cytokines like IL-1β [57], the production of leukotriene B4 (LTB4) by the neutrophils [58] and can modulate T-cell differentiation, and reduce antigen presentation via the major histocompatibility complex class II (MHC-II) [59,60,61]. It is important to underline that, in many of these studies, favorable outcomes were also achieved in control groups. This can be related to the inappropriate use of olive oil or corn oil as vehicle control, which possess their own anti-inflammatory activity. Other limits are the small sample size, a high dropout rate and, with some exceptions, the short duration of the trials.

### 3.3. Polyphenols

One of the key foods in the MD is extra virgin olive oil (EVOO), which contains a multitude of bioactive compounds, including phenolics, sterols, carotenoids and triterpenic alcohols. The EVOO’s content in phenolic compounds is associated with potent antioxidant properties and has been correlated to the wide-ranging health benefits of the MD [62,63,64]. Recently, there has been a growing interest in investigating the potential benefits of EVOO consumption in the context of autoimmune diseases.

Oleocanthal (OL) is a typical phenolic component of EVOO, and recently, it has explored its preventive potential against RA. In a murine model of collagen-induced arthritis, Montoya et al. proved the ability of OL to clinically prevent the disease onset. In particular, the OL-enriched diet-fed group showed reduced levels of joint inflammatory biomarkers, including IL-1β, interferon-γ (IFN-γ), tumor necrosis factor-α (TNF-α), IL-6, IL-17 and matrix metalloproteinases 3 (MMP-3), but also inducible nitric oxide synthase (iNOS), cyclooxygenase-2 (COX-2) and prostaglandin-E2 (PGE2) [65]. The mechanistic analysis revealed the enhancement of nuclear factor erythroid 2-related factor/heme oxygenase-1 (Nrf2/HO-1) and the inhibition of mitogen-activated protein kinases/nuclear factor-κB (MAPKs/NF-κB) signaling pathways. A recent investigation found that EVOO administration prevented the release of nitric oxide (NO) and the production of pro-inflammatory cytokines in a mice model of pristane-induced systemic lupus erythematosus (SLE). The amount of EVOO used was equal to 20 g daily consumption for a person of 70 kg body weight [66]. In agreement with these results, a previous study demonstrated the beneficial effects of EVOO on renal damages and inflammatory biomarkers at splenic levels in the same mice model of SLE [67]. In both cases, the EVOO diet supplementation was compared with a sunflower oil diet supplementation, and both investigations agreed on greater EVOO effectiveness, probably due to its phenolic composition.

Olive derivatives have shown diverse effects on circulating thyroid hormone levels in animal models, including both euthyroid and experimentally induced autoimmune thyroiditis models, with an increase in total T3 levels being the most observed outcome [68]. The review by Pang et al. highlighted that the analysis of the existing literature revealed that olive oil, olive leaf extracts and solid olive residue improved thyroid function in both euthyroid and hypothyroid animals, with the latter also showing improvements in oxidative status [68].

Another key custom within the MD is the moderate consumption, during meals, of wine, particularly red wine. This habit has been associated with the beneficial health effects of MD, most probably due to wine’s phenolic composition [69,70]. In relation to autoimmune disorders, different investigations demonstrated an inverse correlation between moderate alcohol consumption and the frequency of RA [71,72], SLE [73,74] and autoimmune hypothyroidism [17,18,75]. The beneficial health effects of moderate wine consumption have often been linked to a particular phenolic compound called resveratrol, a stilbene found in grape skin. Resveratrol is recognized for its bioactive properties, including anti-cancer, anti-inflammatory, neuroprotective and antioxidant effects [76,77,78] and, recently, several studies evidenced a potential protective effect of this compound in different autoimmune settings. A clinical randomized controlled trial enrolling 100 subjects with RA (68 women and 32 men) demonstrated the potential application of resveratrol as an adjuvant to the classical anti-rheumatic therapies with significant enhancement in the reduction in several inflammatory biomarkers and in the clinical markers and disease activity score [79]. The potentiality of resveratrol as an additional treatment to the traditional medications for RA has been confirmed also in the study from Lomholt et al., where resveratrol synergized with methotrexate [80]. Other investigations concluded that the association of resveratrol with other compounds, like curcumin and sodium alginate, improved its efficiency in different animal models of arthritis, reducing paw edema and the levels of chemokines in the synovial tissue, respectively [81,82].

Studies on human fibroblast-like synoviocytes showed that 50 µM resveratrol was able to suppress PGE2, IL-1β, phosphoinositide 3-kinase/protein kinase B (PI3K/AKT) signaling pathway, COX-2, ROS, NF-κB transcription factor, extracellular signal-regulated kinases (ERK)1/2 and p38 MAPK and MMP-3, resulting in an overall anti-inflammatory effect [83,84]. The anti-rheumatic activity has also been confirmed in fibroblast-like synoviocytes derived from rats administered with 5, 15 and 45 mg/kg doses of resveratrol for 12 days [85]. Interestingly, all these studies used as supplement red grapes-derived resveratrol.

In addition, resveratrol was shown to possess therapeutic potential against RA complications. In a collagen-induced arthritis model, resveratrol was able to lower the rate and the duration of atrial fibrillation, reducing the manifestation of apoptosis and fibrosis and the increase in inflammatory markers levels like IL-6 and TNF-α [86]. Furthermore, resveratrol has shown protective efficacy against SLE, by counteracting CD4^+^ T cells’ activation and B cells proliferation together with a direct effect on kidney involvement (reducing proteinuria, IgM and IgG deposition and lesions) in a pristane-induced mouse model [87], and also displayed vascular protective activity in the ApoE^−/−^ Fas^−/−^C57BL/6 models [88]. Additionally, resveratrol normalized the activity of the cholesterol efflux pathway, demonstrating its anti-atherogenic potential [89,90], which is a common complication of this autoimmune disease.

Overall, available studies to date have highlighted the potential benefits of polyphenols, particularly those derived from EVOO and red grapes, in counteracting the pathogenic mechanisms underlying autoimmune diseases, especially rheumatic ones. However, research on thyroid-related conditions has been less explored in this regard. It is important to note that some studies were conducted on animal models, so confirmation in humans is needed. Further efforts should be made to explore the therapeutic and, more importantly, the preventive potential of these key components of the MD.

## 4. Beneficial Effects of Mediterranean Diet in Autoimmune Diseases: Evidence from Scientific Literature

In recent years, several studies of different intent and design have evaluated the potential benefits of MD in various systemic and organ-specific autoimmune disorders. We focused the present review on rheumatic diseases (like rheumatic arthritis and systemic lupus erythematosus), and thyroid diseases (like Hashimoto’s thyroiditis), since they often occur in the same individuals, mainly in females with increasing age [91,92,93].

### 4.1. Mediterranean Diet and Autoimmune Thyroiditis

Autoimmune thyroiditis, also known as Hashimoto’s thyroiditis, represents a paradigmatic example of an organ-specific autoimmune disorder, and the most frequent autoimmune endocrine disease in the general population. It occurs in genetically susceptible individuals, triggered by an expanding number of environmental factors [17,91]. The loss of tolerance towards thyroid antigens and the subsequent autoimmune reaction leads to tissue inflammation, progressive damage and atrophy, resulting in hypothyroidism [91].

In clinical practice, the coexistence of other autoimmune disorders, either organ-specific or systemic, is a common event in HT patients at any age. In particular, a strong association has been demonstrated between HT and rheumatic diseases, mostly in females at increasing ages [92,93]. Due to the common underlying pathogenetic mechanism, autoimmune disorders may share genetic predisposing factors, but also some environmental triggers. Understanding these associations may help develop common strategies to improve multiple clinical outcomes in these patients. As the incidence and prevalence of autoimmune diseases have been increasing over the last three decades, research pointed to environmental factors as the main drivers of these rapid epidemiological changes [94]. Particular attention was paid to the role of lifestyle factors typical of modern societies, such as reduced physical activity, excess body weight and diet.

Starting from the pioneering observations of Trowell [95], a growing body of evidence pointed to dietary habits as a determinant of risk for the development of thyroid autoimmunity and/or dysfunction, as summarized in Table 1.

In 2013, Tonstad et al. evaluated the prevalence and incidence of thyroid dysfunction among members of the Seventh-day Adventist church, whose eating habits were predominantly based on plant-based foods [96,97]. They found that vegan diets were associated with reduced risk of both hyper- and hypothyroidism compared to omnivorous diets, suggesting a protective role of plant-based diets against thyroid dysfunction [96,97].

In the following years, a growing number of studies have examined how dietary patterns may affect thyroid function parameters and/or autoimmunity markers.

Some of these studies focused on the intake of a single nutrient element [98,99,100,101,102,103], or on the anti-inflammatory potential of the diet, defined on the basis of the frequency intake of foods well-known to have anti-inflammatory properties such as fiber, vitamins and spices [104], rather than on a dietary model. Liu and colleagues investigated the relationship between the dietary inflammatory potential and thyroid function in a large cohort of adult males (2346 subjects) using data from the National Health and Nutrition Examination Survey (NHANES) [105]. They calculated the dietary inflammatory index (DII) based on the consumption of anti-inflammatory foods (such as fiber, vitamin C, flavonoids, garlic and herbs like rosemary and thyme) versus pro-inflammatory foods (including animal fat, carbohydrates and animal protein). The results indicated a positive association between DII and total T4, with subjects consuming a more pro-inflammatory diet showing higher levels of total T4 and total T3—even though these values remained within the normal range—while no consistent effects were observed on fT3, fT4 or TSH. These findings were maintained in a multivariate model adjusted for variables such as age, race, smoking status, energy and protein intake, education level, urinary iodine concentration (UIC) and BMI, with the relationship being more pronounced in obese subjects and those with UIC levels indicating iodine deficiency [105]. Another study by Chen and coworkers [106] assessed the relationship between dietary inflammation and thyroid function in Hashimoto’s thyroiditis patients using the same data source, the NHANES. They found that DII was positively correlated with TSH and total T4, and HT patients with more pro-inflammatory diet habits had higher levels of both TSH and TT4. However, the association with thyroid antibodies was not significant [106]. More recently, Klobučar and coworkers investigated the associations between the inflammatory potential of a diet and thyroid function in a cohort of 149 HT adults diagnosed with Hashimoto’s thyroiditis [107]. They found that HT patients adhering to a more anti-inflammatory diet appeared to have lower TSH levels, higher free T4 levels and lower BMI values. Also, a significant association between the DII and free T4 was reported, suggesting that a more pro-inflammatory diet might negatively impact thyroid hormone levels [107]. In the study, anti-TPO levels were twice as high, but not significantly higher, in patients with a high pro-inflammatory diet [107]. Interpreting these studies is challenging due to the numerous factors influencing circulating thyroid hormones, particularly when both TSH levels and circulating T3 and T4 remain within normal limits. Instead, the described hormone fluctuations in relation to DII might be the consequence of slight modifications in peripheral sensitivity to thyroid hormones or in the binding of thyroid hormones to transport proteins rather than of direct effects on thyroid function. Moreover, two studies rely on registry data, allowing for an analysis of a large number of patients but not an evaluation of any causal inferences.

Noteworthy, the study by Klobučar focused on the importance of dietary composition in modulating the inflammatory potential of the diet, as higher DII scores (indicating more pro-inflammatory diets) were associated with higher intake of proteins and lower intakes of beneficial nutrients and phytochemicals. On this basis, the authors concluded that the best dietary option would be the Mediterranean diet, rich in anti-inflammatory and antioxidant components, which has been known for promoting cardiovascular and metabolic health and reducing inflammation [107].

The potential effects of MD on thyroid function were explored by Zupo et al. The author evaluated adherence to the MD via the PREDIMED questionnaire and thyroid function in 324 overweight or obese euthyroid subjects from Southern Italy [108]. They observed that higher adherence to the MD was associated with lower serum levels of free T3 (fT3) and free T4 (fT4). Further analysis of individual items on the PREDIMED questionnaire revealed that a greater intake of extra-virgin olive oil was linked to reduced levels of both fT3 and fT4. When adjusting for gender, age and BMI using logistic regression, only the association between FT4 and MD adherence remained significant, and no effect on serum TSH was found [108].

Some other studies have specifically focused on the role of dietary patterns in increasing the risk of thyroid autoimmunity, independent of thyroid dysfunction. In the study by Alijani and coworkers, the DII was positively correlated with the levels of TSH and autoantibodies TPOAb and TGAb, whereas the dietary total antioxidant capacity was negatively correlated with antibody levels [109]. Normal BMI and daily fruit and vegetable consumption all contribute to maintaining oxidative stress at low levels in HT patients. When evaluating dietary habits, low oxidative stress was associated with eating vegetables at least seven times a week and fruits at least 14 times a week [110]. In 2020, Kaličanin and colleagues used a food frequency questionnaire (FFQ) to compare food group consumption among Hashimoto’s thyroiditis (HT) patients and healthy controls, with particular attention to fat intake and the choice between vegetable and animal fats [111]. Their results showed that HT patients consumed more animal fat and processed meat than controls, who instead ate more non-processed red meat, non-alcoholic beverages, whole grains and plant oils. A subgroup analysis revealed that HT patients undergoing levothyroxine (LT4) therapy consumed more red meat than those not receiving substitutive treatment. Additionally, the study found that HT patients did not significantly change their dietary habits following diagnosis, suggesting that nutritional aspects are often overlooked by both patients and their physicians [111]. In 2021, Ruggeri et al. assessed the dietary habits of a cohort of euthyroid subjects from Southern Italy, identifying significant differences between HT patients and healthy individuals [13]. Specifically, HT patients had higher intakes of animal-derived foods—including both fresh and processed meats, dairy products, fish—and commercial sweetened products, while healthy controls consumed more vegetables, legumes and nuts. Importantly, there were no differences in body weight or BMI between HT subjects and controls, with most participants being of normal weight. Adherence to the MD, as measured by the PREDIMED questionnaire, was lower in HT patients compared to controls. Furthermore, in a multivariate logistic regression model, the PREDIMED score independently predicted thyroid autoantibodies positivity, suggesting a protective effect of the MD against thyroid autoimmunity [13].

More recently, a study aimed at evaluating lifestyle changes across the pandemic period demonstrated that subjects diagnosed with thyroid diseases (70% autoimmune) displayed a higher prevalence of excess weight, lower adherence to the MD and reduced physical activity levels compared to healthy controls from the same geographical area in Italy. Despite the different intent, the study was confirmatory of previous evidence in favor of the role of the MD in counteracting thyroid autoimmunity and related dysfunction [112].

An interventional study was performed on a cohort of 40 Egyptian female patients diagnosed with HT and already under L-T4 therapy [113]. A modified Mediterranean dietary plan for 12 weeks achieved statistically significant reductions in both autoantibodies and TSH levels (*p* < 0.01), whereas both fT3 and Ft4 levels significantly increased. A significant BMI reduction was also achieved (*p* < 0.01). The authors concluded that MD may represent a complementary therapy for Hashimoto’s thyroiditis, achieving lower disease activity, improved thyroid function as well as weight reduction [113].

Despite the limitations of these studies, which need confirmation on large series in randomized controlled trials, promoting adherence to the MD could offer a safe, accessible and cost-effective approach to better disease control.

**Table 1:** Summary of the clinical studies addressing the relationship between dietary habits and thyroid functional status and/or autoimmunity.

Reference	Disease Type	Study Type	Number of Participants, Age, Gender Distribution	Diet Effects	Intervention	Summary of Results
Tonstad et al., 2013 [96]	Hypothyroidism	Observational study	65,981 subjects belonging to the Seventh-day Adventist church aged >30 years	Positive	Comparison between vegetarians and omnivorous subjects	Reduced risk of prevalent (OR 0.89, 95% CI: 0.78–1.01) and incident (OR 0.78, 95% CI: 0.59–1.03) hypothyroidism in vegetarians
Tonstad et al., 2015 [97]	Hyperthyroidism	Observational study	65,981 subjects, members of the Seventh-day Adventist church aged >30 years	Positive	Vegetarian diets vs. omnivorous diets	Reduced risk of prevalent hyperthyroidism in vegan (OR = 0.49; 95% CI 0.33, diets compared to omnivorous diets
Zupo et al., 2020 [108]	No diagnosed thyroid disease	Observational study	324 euthyroid overweight/obese subjects from Southern Italy (228 F and 96 M, aged 14–72 years)	Uncertain	Assessment of MD adherence with the PREDIMED questionnaire Assessment of EVOO consumption	Inverse correlation between the MD score and serum fT3 (*p* < 0.01) and fT4 (*p* < 0.01) levels; no correlation with serum TSH levels. MD was associated with a slightly reduced thyroid function, within normal limits
Liu et al., 2021 [105]	General population	Cross-sectional study	2346 male subjects from U.S. aged ≥ 20 years (data from NHANES)	Positive	Dietary inflammatory index (DII) score	Positive correlation between DIP and serum total T4 levels (*p* = 0.0044); no association with serum freeT3, free T4 or TSH levels
Chen et al., 2023 [106]	HT	Cross-sectional study	964 subjects, 67.6% females. Mean age: 51.4 ± 16.2 years	Positive	Assessment of the relationship between dietary inflammation and thyroid function	Positive correlation between DII and TSH and total T4 levels
Alijani et al., 2024 [109]	HT	Hospital-based case–control study	230 participants (115 HT patients, 54.5% females and 115 controls, 5.5% females)Mean age 39.76 ± 9.52 years	Positive	Assessment of the relationship between DII and DTAC and thyroid autoantibodies	In the HT group, the DII level was higher (*p* < 0.001) and the DTAC level was lower than those in the healthy group (*p* = 0.047) DII had a positive correlation with TPOAb, TGAb and TSH levels, while DTAC had a negative correlation with anti-TPO and TG-Ab (*p* < 0.050).
Giannakou et al., 2018 [110]	HT	Observational study	218 euthyroid HT women with HT (102 with thyroxine replacement and 114 without) mean age 46.0 ± 12.7 years	Positive	Serum TOS measurement in relation to BMI and dietary habits	Low fruit and vegetable consumption and high BMI were associated with high TOS
Kaličanin et al., 2020 [111]	HT	Observational study	924 adult subjects: 491 HT patients (93% female, median age 38 years) and 433 controls (60% females, median age 51 years)	Positive	Assessment of consumption frequency of food groups	Increased consumption of animal fat (*p* < 0.0001) and processed meat (*p* = 0.0012) in HT pts. Increased consumption of red meat (*p* < 0.0001), non-alcoholic beverages (*p* < 0.0001), whole grains (*p* < 0.0001) and plant oil (*p* < 0.0001) in controls association of plant oil consumption with increased fT3 levels in HT patients (*p* < 0.0001)
Ruggeri et al., 2021 [13]	HT	Observational study	200 subjects: 81 (71 F) HT pts and 119 (102 F) controls from Southern Italy, median age, 37 years	Positive	Assessment of adherence to the MD with the PREDIMED questionnaire. Assessment of consumption frequency of food groups	Increased intake frequencies of animal foods (meat, *p* = 0.0001; fish, *p* = 0.0001; dairy products, *p* = 0.004) in HT pts Increased intake frequencies of plant foods (legumes, *p* = 0.001; fruits and vegetables, *p* = 0.030; nuts, *p* = 0.0005) in controls Lower adherence to the Mediterranean diet in HT pts compared to controls (*p* = 0.0001) PREDIMED score was an independent predictor of TPOAb positivity (OR 0.192, 95% CI 0.074–0.500, *p* = 0.001)
Klobučar, S et al., 2024 [107]	HT	Observational Cross–Sectional Multicenter Study	149 adults diagnosed with HT (140 females) aged 19 to 72 years	Positive	Assessment of DII using a 141-item FFQ Measurement of TFTs and autoantibodies	After adjustment for potential confounders, DII was positively correlated with TSH (*p* = 0.002) and BMI (*p* = 0.04)
Corrias et al., 2024 [112]	HT	Observational study	116 patients (96 F, aged 57.2 ± 13.1 years) affected by thyroid disorders (70%, autoimmune thyroid disease), all euthyroid, and 248 healthy adults (65% F, aged 53.1 ± 12.1), from Sardinia, Italy	Positive Neutral on QuoL	Assessment of adherence to the MD by means of both MDS and PREDIMED questionnaires Physical Activity Level (PAL), and Quality of Life (SF-12) were also assessed	Lower adherence to the MD (*p* = 0.003) and a significant increase of sitting time (*p* < 0.001), along with a not significantly reduced PAL, were reported in patients compared to healthy subjects No differences in QuoL scores
Shady et al., 2024 [113]	HT	One-arm clinical trial	40 female patients with HT, under L-Thyroxine therapy	Positive	Measurement of TFTs, thyroid autoantibodies and BMI at baseline and after 12 weeks of a modified MD plan.	Significant reductions in autoantibodies, TSH, fT3 and fT4 levels (*p* < 0.01) Significant reduction in BMI (*p* < 0.01)

Abbreviations: TSH: thyroid stimulating hormone or thyrotropin; TT4: total thyroxine; fT4: free thyroxine; fT3: free triiodothyronine; TFTs: thyroid function tests; TPOAb: anti-thyreoperoxidase antibodies; HT: Hashimoto’s thyroiditis; MD: Mediterranean Diet; PREDIMED: PREvención con DIeta MEDiterránea; MDS: Mediterranean Diet Score; FFQ: food frequency questionnaire; DTAC: dietary total antioxidant capacity; DII: dietary inflammatory index; BMI: body mass index; EVOO: extra-virgin olive oil; NHANES: National Health and Nutrition Examination Survey; QuoL: quality of life; SF-12: the 12-Item scale of the Short Form Survey for quality of Life assessment; PAL: physical activity level; TOS: total lipid peroxide levels.

### 4.2. Mediterranean Diet and Rheumatic Diseases

#### 4.2.1. Mediterranean Diet and Rheumatoid Arthritis

RA is a chronic autoimmune disease characterized by symmetrical synovitis of the peripheral joints and systemic inflammation leading to multiple organ damage. Synovitis is the main feature of the disease and, in the most severe cases, can lead to cartilage and bone erosion with progressive destruction of the joint.

The development of RA is multifactorial. Risk factors include genetic susceptibility due to polymorphic variants of several genes involved in the immune response, such as *HLA-DRB1*, *OLIG3/TNFAIP3*, *PTPN22*, *STAT4* and *TRAF1-C5*, environmental factors such as cigarette smoke or infections, lifestyle and hormones.

The pathogenesis of RA is complex. The latest theories assume that post-translational modifications of self and foreign (e.g., bacterial) proteins play a decisive role in the development of the disease [114]. The most important post-translational modification of proteins associated with RA risk is citrullination. Citrullination is a process that may precede the onset of clinical symptoms and manifestations and characterize more aggressive forms of RA when it affects key proteins involved in the maintenance of connective tissue microarchitecture. Citrullination of intrinsic proteins may favor the generation of autoantigens and thereby trigger autoreactivity. Citrullinated proteins are produced in the airways and buccal mucosa in response to cigarette smoking or periodontal infections. At these sites, antigen-presenting cells (APCs) can be activated and induced to migrate and present citrullinated self-peptides to T lymphocytes in the lymphoid organs. The T lymphocytes can then differentiate into effector cells, memory cells or Treg cells. Autoreactive T cells can further promote the activation and proliferation of B lymphocytes and the final development to the plasma cell stage with the production of autoantibodies. Plasma cells can, therefore, produce anticitrullinated peptide antibodies (ACPAs) [115], which are pathognomonic of RA, or other autoantibodies such as rheumatoid factor (RF), antinuclear antibodies (ANA) or anti-RA33 antibodies. Antibodies bound to their antigens can, in turn, activate macrophages, which are important effector cells and play a central role in the pathogenesis of RA. Macrophages can also be activated by T lymphocytes and produce various systemic cytokines, such as TNF-α, IL-1 and IL-6, which collectively contribute to local and systemic inflammation and damage [116].

Clinically, RA is characterized by symmetrical and bilateral involvement of the small joints, especially the hands, wrists and feet. Other affected joints include the knees, elbows and ankles. The spine is usually not affected, with the exception of the cervical spine. RA patients suffer from joint pain, swelling and stiffness, which can be accompanied by general malaise and chronic fatigue. RA can also affect the lungs, leading to pulmonary nodules or interstitial lung disease (ILD), the skin with the formation of rheumatoid nodules, the kidneys, the eye and the peripheral nervous system (PNS) [117]. It is important to note that RA is associated with heart disease, particularly in terms of increasing cardiovascular risk. It is estimated that RA patients have a 1.5 to 2-fold increased risk of developing cardiovascular events [118]. This is particularly true for patients with long-standing disease, high titers of autoantibodies and other extra-articular manifestations [117].

The treatment of RA has made remarkable progress with the formulation of increasingly specific and appropriate drugs that can intervene in certain phases of immune system activation. Nowadays, clinicians can choose between conventional synthetic disease-modifying antirheumatic drugs (csDMARDs), glucocorticoids, biologic (b) DMARDs and targeted synthetic (ts) DMARDs to personalize the therapeutic strategy and achieve sustained clinical remission [119]. Unfortunately, international guidelines for this type of patient usually do not provide specific dietary recommendations. A healthy and balanced diet can be a protective factor as it reduces the risk of developing cardiovascular or metabolic diseases. As mentioned above, several preclinical and clinical studies indicate that single components of MD can play a positive role in combating RA inflammation [51,52,53,54,65,73,80]. In addition, recent evidence suggests that changes in the gut microbiota may be correlated with an increase in RA disease activity. Changing the dietary habits of patients could, therefore, have a positive effect on the inflammatory state associated with the disease [49]. Several studies have investigated the efficacy of different dietary patterns, including the MD, in the treatment of RA patients (Table 2).

The randomized clinical trial (RCT) by Papandreou et al. showed that RA patients randomly assigned to the MD group, in conjunction with physical activity for 12 weeks, had a significant reduction in Disease Activity Score on 28 joints (DAS28) compared to the control group and also had an improvement in cardiometabolic outcomes such as body weight, body composition, blood glucose and concentration of vitamin D [120]. Similar results regarding the reduction in disease activity assessed with the DAS28 were also obtained in other RCTs, e.g., by Sköldstam et al. and Abendroth et al. [26,49]. In the study by Sköldstam et al., the patients were randomly divided into two groups: the MD group and the control group, which followed a typical Western diet. The study reported significant improvements in physical function in the MD group, in addition to a reduction in DAS28 [26]. In the study by Abendroth et al., the patients were instead assigned to the MD group or a 7-day fasting therapy. Both groups achieved a similar reduction in disease activity scores as assessed by the DAS28 compared to baseline. Another endpoint of the study was the reduction in musculoskeletal pain, as assessed by the Visual Analog Scale (VAS). In this regard, a more significant reduction in pain was observed in the patients who underwent a 7-day fasting therapy than in the patients in the MD group [49]. Another study conducted on 44 RA patients who followed either the MD or Healthy Eating Guidelines (HEG) for 12 weeks showed that adherence to the MD improved physical function and quality of life, as assessed by the validated Health Assessment Questionnaire-Disability Index (HAQ-DI) and Rheumatoid Arthritis Quality of Life (RAQoL) questionnaires, compared to the HEG intervention [121].

The benefits of the MD can be enhanced by an active lifestyle. Two RCTs examined the efficacy of the MD and a dynamic exercise program (DEP) in patients with RA. In the first study by Pineda-Juárez et al., 34 patients were randomly assigned to an MD + DEP intervention, while 34 patients were assigned to DEP alone and 38 patients to MD alone for 24 weeks [122]. The DEP intervention consisted of an eighty-minute exercise program performed twice a week aimed at improving muscle strength, range of motion and joint flexibility. In the second 24-week study by García-Morales et al., 36 RA patients participated in the MD + DEP group, while 37 were assigned to the DEP alone group, 40 to the MD alone group and 31 were enrolled as a control group [123]. The DEP intervention consisted of training twice a week for 80 to 90 min. In both studies, the combination of DEP and MD led to an improvement in health-related quality of life (HRQoL) and a decrease in the disability index (HAQ-DI).

To summarize, RA patients can benefit from the MD in terms of both disease activity and disease-related complications. Due to its anti-inflammatory and antioxidant effects and its role in maintaining body weight, the MD could be a useful adjunct in the treatment of RA. Although the overall scientific evidence supports this view, further studies are needed.

#### 4.2.2. Mediterranean Diet and Spondyloarthritides

Spondyloarthritides (SpAs) are a heterogeneous group of inflammatory diseases linked by epidemiologic, genetic, clinical and radiologic features. The main clinical manifestation of SpAs is inflammation of the spine, which may be associated with peripheral arthritis, enthesitis, dactylitis and extra-articular manifestations such as psoriasis, uveitis and colitis.

SpAs include several subtypes, such as ankylosing spondylitis (AS), non-radiographic axial SpA (nr-axSpA), psoriatic arthritis (PSA), arthritis associated with inflammatory bowel disease (IBD), reactive arthritis (ReA) and undifferentiated forms [124]. Epidemiologically, there is no difference between the overall prevalence of SpA in the two sexes, but axial SpA predominantly affects males (M/F 3/1). The average age of onset for axial SpA is between 26 and 45 years, whereas other forms of SpA may occur later [125]. The pathogenesis of SpA is not yet fully understood. As in RA, SpA develops as a result of an interaction between a favorable genetic substrate, dysbiosis, infections and other environmental factors. The genetic predisposition is reflected in the frequency of the HLA-B27 allele in more than 90% of AS patients. Being a carrier of such an allele is associated with a 20 to 44-fold risk of developing SpA. Various theories have attempted to explain the role that HLA-B27 plays in triggering the disease. One of these theories is based on a “molecular mimicry” mechanism, according to which some arthritogenic antigens derived from pathogenic species can more easily bind to the MHC class I encoded by HLA-B27 and thus be presented to cytotoxic T lymphocytes, triggering an immune response at the level of bone and cartilage. Another theory is based on a “misfolding” of proteins in the endoplasmic reticulum, according to which the heavy chain of HLA-B27 in the rough endoplasmic reticulum would undergo an altered folding with homo-dimerization, leading to the activation of the unfolded protein response (UPR), autophagy and the production of IL-23. According to another theory, HLA-B27 on the plasma membrane could target the killer immunoglobulin receptor (KIR) expressed by T and natural killer (NK) cells, which would activate the production of IL-17 regardless of the presence of an exogenous antigen [126]. HLA-B27 may also influence the composition of the gut microbiota and thus contribute to the maintenance of the dysbiosis observed in SpA patients. The qualitative and quantitative alteration of the gut microbiome in the presence of a leaky gut barrier may lead to an interaction between bacterial peptides and leukocytes. This event could lead to an abnormal inflammatory response and eventually to the production of IL-23 and IL-17 [127]. Finally, patients with SpA show an altered response to mechanical stress at the entheses, which are the bony attachment points of tendons, ligaments and fasciae. Repeated microtrauma to the enthesis in genetically predisposed individuals may induce the abnormal production of proinflammatory cytokines such as IL-23 and adhesion molecules by resident myeloid cells, leading to local recruitment of inflammatory cells. This process, known as enthesitis, is a pathognomonic feature of SpA. Chronic enthesitis can eventually lead to bone remodeling with phenomena of bone neoapposition and ankylosis [128]. All in all, the proposed pathogenic mechanisms in SpA converge on the overproduction of cytokines such as TNF-α, IL-17 and IL-23. Therefore, pharmacological treatment of these patients mainly focuses on the use of bDMARDs, which can neutralize these cytokines and suppress the downstream inflammatory cascades [129]. Conversely, the use of csDMARDs appears to be less useful in the axial forms and is limited to the peripheral manifestations.

SpA are an example of the rheumatic diseases for which the strongest association with gut dysbiosis has been established. As the MD has numerous anti-inflammatory properties and may remodel the gut flora, it could help to reduce inflammation in SpA [130]. The Italian Society of Rheumatology (SIR) emphasizes that a healthy diet, together with physical activity, complements pharmacological therapies in the treatment of rheumatic diseases [131]. However, there are no specific guidelines that deal exclusively with the MD in the context of SpA.

A search of the PubMed database found four relevant articles dealing with the effects of MD in SpA. Three of them reported such outcomes in PSA patients and one in axSpA patients (Table 3).

In a randomized crossover study conducted on 26 patients with PsA and psoriasis (PsO), the effects of MD and the ketogenic diet on these diseases were investigated. Both diets led to a significant reduction in weight, body mass index (BMI), waist circumference, total and visceral fat mass, but the ketogenic diet also led to a significant reduction in PsO area and PsO area severity index (PASI), Disease Activity Index for PsA (DAPSA) and the levels of IL-6, IL-17 and IL-23, which were not observed after the MD [132]. Another multicenter cross-sectional observational study investigated adherence to the MD in 211 patients with PsA and its effects on disease activity. The results showed that in PsA patients, higher disease activity, as measured by DAPSA, was correlated with lower adherence to the MD, suggesting a potential anti-inflammatory benefit of this dietary pattern [134]. Finally, the influence of the MD and physical activity on the presentation and severity of PsA was investigated in a cross-sectional observational study. PsA disease activity, as measured by DAPSA, tender and swollen joint count, erythrocyte sedimentation rate (ESR) and PASI were inversely related to the level of physical activity performed. Greater adherence to the MD was associated with a reduction in ESR, PASI and body surface area (BSA) indices. After adjustment for BMI, physical activity maintained its negative correlation with PsA activity, while diet had a significant correlation only with enthesitis [29]. Regarding axSpA, an observational study conducted on 110 patients showed an improvement in disease activity as measured by Ankylosing Spondylitis Disease Activity Score with CRP (ASDAS-CRP), which was more frequent in the group of patients following a MD pattern than in the control group [28].

In summary, the evidence for the benefit of MD in SpA is limited due to the very small number of trials and the study design. However, given the central role of gut dysbiosis in the pathogenesis of SpA, it is plausible that dietary interventions consisting of recommending foods with a low pro-inflammatory profile, such as those found in the MD, could improve clinical manifestations and disease progression. The combination with physical activity could be even more effective and have a synergistic effect with concomitant medications.

### 4.3. Mediterranean Diet and Connective Tissue Diseases

Connective tissue diseases (CTDs) are a group of chronic autoimmune diseases with heterogeneous manifestations. They are characterized by a complex and polyhedral im-munopathogenic scenario with the production of autoantibodies of different specificities that may encounter their antigens in different organs and tissues or form circulating immune complexes, whose final deposition in small vessels of the affected body regions may activate further immunological cascades. The final result is systemic and implies local inflammation, tissue damage, abnormal fibrotic repair and loss of function of affected organs. CTDs have a multifactorial origin, with the interaction of genetic and environmental factors contributing to their development [134]. Among the most peculiar CTDs are SLE, systemic sclerosis (SSc) and pSS. Although each disease has its own clinical, laboratory and instrumental features, they may share common risk factors, antibody patterns, and clinical manifestations that make it possible for two or more diseases to overlap in the same individual.

#### 4.3.1. Systemic Lupus Erythematosus

SLE is the most important CTD. The disease is characterized by a relapsing–remitting course [135], mostly affects women of fertile age and has an estimated incidence of 0.3–23.2 cases/100,000 person–years, which varies by geographic region [136]. SLE encompasses a broad spectrum of clinical manifestations that can affect the skin, blood, heart, lungs, kidneys, central and peripheral nervous system and musculoskeletal system. In addition, SLE patients have systemic symptoms such as fever and fatigue. The organ damage is mediated by the activation of the innate or acquired immune response. The main players in this scenario include B and T lymphocytes, plasmacytoid dendritic cells (pDC) and neutrophils, which produce various cytokines and autoantibodies that in turn can be deposited in vessels and form immune complexes. Of these, the most important for the diagnosis of the disease are anti-nuclear antibodies (ANAs), anti-double-stranded DNA antibodies (anti-dsDNAs) and anti-Smith antibodies (anti-Sm). The therapeutic management of SLE is based on a complex algorithm that uses various traditional or biological immunosuppressive agents to achieve remission or low disease activity [137]. Unfortunately, the international guidelines remain unspecific regarding the treatment of SLE with non-pharmacological measures, including diet. It is important to emphasize that SLE is an independent risk factor for cardiovascular disease, which may depend on both disease activity and therapies [137]. SLE patients may, therefore, benefit from the MD both in terms of disease activity and disease-related complications [73,74,87,88]. However, the evidence available in the literature shows limited and controversial data. It is uncertain whether a healthy diet can prevent the risk of developing SLE. In a large cohort study of more than 100,000 women, 194 of whom were incidentally diagnosed with SLE, there was no association between the likelihood of an SLE diagnosis and dietary habits, including the MD, although a high intake of nuts or legumes showed an inverse correlation with SLE risk [138]. Similarly, there is only weak evidence that adherence to the MD can improve SLE domains or reduce SLE-associated cardiovascular risk. In a cross-sectional study of 280 SLE patients, a significant association was found between adherence to the MD and disease activity or increase in damage measured with the Systemic Lupus Erythematosus Disease Activity Index 2000 (SLEDAI-2K) and SLICC/ACR Damage Index (SDI) [31]. However, the effects of dietary changes in SLE patients may be less pronounced than those of exercise. In a cross-sectional study conducted on 145 SLE patients, 49 of whom maintained a healthy lifestyle, physical activity was the most important factor for improving various SLE domains, including fatigue, depression or anti-dsDNA antibody titers, compared to smoking and MD adherence [139]. Furthermore, a Spanish study that cross-sectionally examined adherence to the MD and the presence of metabolic syndrome (MetS) and associations with SLEDAI-2K and SDI scores in a cohort of 293 SLE patients found no significant associations with MD adherence, while MetS significantly affected SDI scores and complement levels [140]. In a cross-sectional study of 76 SLE women, no association was found between MD adherence and arterial stiffness, inflammation or use of corticosteroids or immunosuppressants. However, the authors found a positive correlation between higher consumption of dairy products or lower consumption of red wine and lower use of disease-related medications [141]. Finally, a recent systematic review analyzing 15 studies on diet in SLE showed that a low-fat diet and MD can reduce cardiovascular risk, although large intervention studies are lacking [142].

To summarize, the evidence for the beneficial effects of the MD in SLE is still limited and controversial as there are only a few studies, most of which have a cross-sectional design.

#### 4.3.2. Other CTDs: Sjögren’s Syndrome and Systemic Sclerosis

SS is a CTD characterized by a multisystemic inflammation that particularly affects the salivary glands and lacrimal glands and causes their hypofunction. The immunopathogenesis of SS shares some similarities with SLE: in both diseases, there is an abnormal activation of T and B lymphocytes with subsequent production of many types of autoantibodies that can target specific organs or form immune complexes. Therefore, SS can occur as a primary form (pSS) or be associated with other autoimmune diseases, of which SLE, SSc and RA are among the most common [143]. The incidence of the primary form is 3.9–5.3 per 100,000 person–year among Caucasians, mostly women, while secondary forms are probably more common [143]. The diagnosis of pSS is based on the application of a number of different classification criteria that take into account the positivity of specific autoantibodies (anti-SSA/Ro and anti-SSB/La antibodies), evidence of focal lymphocytic sialadenitis on salivary gland biopsy and instrumental tests to measure salivary and lacrimal secretion [144,145].

SSc is a CTD that has a unique pathogenesis and evolution. The disease is characterized by the triad of inflammation, vasculopathy and fibrosis that contribute to the symptoms and clinical manifestations [146]. The pathogenesis involves endothelial dysfunction leading to progressive occlusion of terminal vessels, production of specific autoantibodies and final activation of myofibroblasts and fibroblasts in vessels, skin and internal organs. These events are clinically reflected in Raynaud’s phenomenon, digital ulcers, pulmonary arterial hypertension and fibrosis of the skin, lungs, intestine and heart. The incidence of SSc varies according to epidemiological studies from 0.77 per 100,000 person–years in the Netherlands to 5.6 per 100,000 person–years in the USA [147]. In this case, too, the disease predominates in women.

Although with different approaches, SS and SSc are both treated with immunosuppressive agents and supportive medication [148,149,150,151]. Scientific evidence on non-pharmacologic strategies, including specific dietary habits, is lacking or of very low quality [7]. Regarding the MD, only two and one studies have investigated the impact on clinical activity of pSS and SSc, respectively. A study published in 2020 with 133 pSS patients eligible showed an inverse correlation between adherence to MD and the likelihood of developing the disease. Eating 1–2.5 servings of fish per week compared to eating less had the strongest protective effect. In multivariate analysis, a lower risk of pSS was associated with a higher intake of galactose, vitamin A retinol equivalents and vitamin C [152]. In an Italian study conducted on 93 Italian pSS patients, fish consumption was also positively associated with a lower prevalence of hypertension. Although the authors found no significant differences in disease activity or cardiovascular risk factors depending on the degree of MD adherence, the 14-item PREvencion con DIeta MEDiterranea (PREDIMED) score was inversely associated with disease activity on the European League Against Rheumatism (EULAR) SS disease activity index (ESSDAI) and the ClinESSDAI [31].

Adherence to the MD in SSc was instead investigated in a recent cross-sectional study. The results showed that only 14.7% of participants adhered optimally to the MD. Poor adherence to the MD was associated with depressed mood and a higher perception of Raynaud’s phenomenon and digital ulcers [32].

Therefore, the current limited data seem encouraging when it comes to promoting adherence to MD in patients with CTDs other than SLE. The results of the studies that investigated the effects of adherence to the MD in CTD patients are summarized in Table 4.

## 5. Conclusions and Perspectives

In conclusion, the MD appears to be a promising complementary tool for managing rheumatic and thyroid autoimmune diseases by potentially reducing disease activity, improving autoimmunity-related outcomes and promoting weight loss. Its beneficial nutritional profile helps enhance immune system performance, maintain a healthy gut microbiota and preserve redox balance, thanks to its antioxidant, anti-inflammatory and immunomodulatory properties.

Rheumatic diseases characterized by inflammation of the joints and connective tissue can benefit from a dual intervention combining the MD with exercise. Indeed, studies comparing dietary patterns with physical activity have shown that exercise achieves better results compared to diet in RA, PsA and SLE [30,122,123,139]. In addition, a fasting or ketogenic diet appears to be superior to the MD in arthritis, although data are limited [49,132]. Both intermittent fasting and the ketogenic diet, which is based on a high fat intake, are known for their antioxidant effects by improving mitochondrial respiration [151,152]. Moreover, they can downregulate the expression of histone deacetylases (HDACs) and the transcription factor nuclear factor kappa B (NF-κB), thus preventing proinflammatory pathways. It is currently unknown whether patients with different autoimmune diseases or at a different stage of disease can benefit from personalized diets or changing dietary patterns (and thus nutrient intake) according to clinical presentation or disease activity. Some studies that investigated MD adherence in RA patients included only individuals with early disease or low disease activity [26,120,123], suggesting that the effects of the MD may be more pronounced in less compromised cases. Studies on SLE are more controversial as adherence to MD may have a neutral effect on disease risk and some of its components such as red wine may even be associated with high intake of immunosuppressants [139,142]. Such results may be attributed to the complexity of assessing whole organ involvement in SLE, as well as concomitant comorbidities and adverse events during treatment that may confound the final results. In addition, the evidence for SLE and other CTDs comes from low-quality studies, highlighting the need for further research in this area. With regard to autoimmune thyroid diseases, the available data clearly indicate a protective role of the MD against oxidative damage, inflammation and glandular dysfunction. A healthy diet in association with physical activity is also useful to prevent weight gain and obesity, well-known to be associated with an increased risk of thyroid autoimmunity.

While current studies suggest that the MD pattern may offer protective effects against autoimmunity, the overall body of evidence remains limited, and more research is needed. Furthermore, well-designed studies, particularly randomized controlled trials, will make it possible to obtain specific recommendations and advice for patients suffering from autoimmune disorders to follow in their daily diets. They will also provide reliable and handy tools to measure the effectiveness of dietary interventions. Nevertheless, there are enough data to support the role of the MD as a healthy food model in the setting of chronic autoimmune disorders. Encouraging adherence to the MD could serve as an effective, cost-efficient lifestyle approach to reduce the burden of autoimmune disorders in modern societies. How the diet could be combined with other treatment approaches, such as drugs, exercise or stress management, to maximize treatment effectiveness, remains to be defined.

## Figures and Tables

**Table 2:** Effects of MD on patients with RA.

Reference	Disease Type	Study Type	Number of Participants, Age, Gender Distribution	Diet Effects	Intervention	Summary of Results
Sköldstam et al., 2003 [26]	RA	RCT	51 patients with RA disease duration of at least 2 years, of which 21 women and 5 men aged 58 (33–73) years in the intervention group and 20 women and 5 men aged 59 (35–75) years in the control group	Positive	26 patients assigned to MD group, 25 assigned to the control group. Five patients excluded from the final analysis	Significant reduction in DAS28 in the intervention group compared to the control group from baseline and improvement in physical function as assessed by HAQ
Papandreou et al., 2023 [120]	RA	RCT	40 women, aged 34.03 ± 5.45 years, with RA for at least 2 years (DAS28 < 3.2)	Positive	20 patients assigned to MD + physical activity for 12 weeks, 20 patients assigned to the control group (usual care)	Significant reduction in DAS28 compared to the control group from baseline and improvement in cardiometabolic outcomes (body weight, body composition, blood glucose, vitamin D concentration)
Raad et al., 2024 [121]	RA	RCT	44 adults (87.5% females), mean age 47.5 ± 10.9 years	Positive	22 patients assigned to the MD group and 22 patients to the HEG group for 12 weeks	Improved physical function, assessed with HAQ-DI, and quality of life, assessed with RAQoL, in patients in the MD group compared to the HEG group
Abendroth et al., 2010 [49]	RA	Prospective observational, non-randomized, clinical trial	50 patients, aged 55.7 ± 7.2 years (95.5% females) in the fasting group and 60.0 ± 12.1 years (92.9% females) in the MD group	Neutral	28 patients assigned to the MD and 22 patients to a 7-day fasting therapy	Reduction in DAS28 in both groups from baseline with similar improvements in both study groups. Significant reduction in VAS pain scores on day 7 after fasting therapy compared to MD
Pineda-Juárez et al., 2022 [122]	RA	RCT	106 women aged 49.5 ± 13.6 years in the DEP + MD group, 47.1 ± 11 years in the DEP group and 48.2 ± 13.2 years in the MD group	Neutral–Positive	34 patients assigned to the MD + DEP group, 34 patients assigned to the DEP group, 38 patients assigned to the MD group for 24 weeks	Increase in handgrip strength in the DEP group Decrease in weight and waist circumference in the MD group. Decrease in HAQ-DI values in the MD + DEP group
García-Morales et al., 2020 [123]	RA	RCT	144 women, aged 51.4 ± 12.4 years in the MD + DEP group, 49.7 ± 11.4 years in the DEP group, 46.3 ± 13.1 years in the MD group, and 49.1 ± 12.1 years in the control group; DAS28 < 3.2	Positive	36 patients assigned to the MD + DEP group, 37 patients assigned to the DEP group, 40 patients assigned to the MD group and 31 patients assigned to the control group (no additional intervention) for 24 weeks	Improvement in HRQoL scores in patients in the MD + DEP group compared to the other groups

Abbreviations: DAS28, Disease Activity Score on 28 joints; DEP, dynamic exercise program; HAQ, Health Assessment Questionnaire; HAQ-DI, Health Assessment Questionnaire-Disability Index; HEG, Healthy Eating Guidelines; HRQoL, Health-Related Quality of Life; MD, Mediterranean diet; RA, rheumatoid arthritis; RAQoL, Rheumatoid Arthritis Quality of Life; RCT, randomized controlled trial; VAS, Visual Analog Scale.

**Table 3:** Effects of MD on patients with SpA.

Reference	Disease Type	Study Type	Number of Participants, Age, Gender Distribution	Diet Effects	Intervention	Summary of Results
Lambadiari et al., 2024 [132]	PsA and PsO	Randomized crossover trial	26 patients, mean ± SD age 52.93 ± 7.33 years, females 75%	Neutral	Patients randomly assigned to the MD or a ketogenic diet arm for 8 weeks. After a washout interval of 6 weeks, the groups were crossed over and observed for a further 8-week period	Significant reduction in PASI and DAPSA scores and IL-6, IL-17 and IL-23 levels after the ketogenic diet. No significant difference in the arm assigned to the MD
Caso et al., 2020 [133]	PsA	Observational multicenter cross-sectional study	211 patients (females 62.09%) with median age of 55 (48–62) years and disease duration of 76 (36–120) months. 27.01% of patients classified as having MetS	Positive	Assessment of PsA disease activity using the DAPSA and CPDAI scores, assessment of MetS and adherence to the MD using the PREDIMED questionnaire	Inverse correlation between the degree of adherence to MD and DAPSA scores
Katsimbri et al., 2024 [29]	PsA and PsO	Observational cross-sectional study	355 patients (279 with PsA and 76 with PsO), with median age of 55 (45.1–62.9) years; 56.6% females	Positive	Assessment of disease activity by DAPSA, LEI, ASDAS, BASDAI, BSA, PASI and HAQ tools. Assessment of MD adherence using the PREDIMED questionnaire and assessment of physical activity using the Short Last 7 Days Self-Administered Format of the International Physical Activity Questionnaire	Inverse correlation between DAPSA score, tender and swollen joint count, ESR values and PASI scores with physical activity levels. Significant correlation between higher MD adherence and lower ESR values, PASI and BSA scores. After adjustment for BMI, significant correlation between physical activity and PsA disease activity and between diet and enthesitis
Ometto et al., 2021 [28]	axSpA (with or without PsO)	Observational monocentric study	110 patients who completed the study, of which 40% females; mean age 51.7 ± 1.3 years	Positive	47 patients who followed MD and 63 patients who did not follow a special diet for 6 months	Significant improvement of the ASDAS-CRP score (≥20%) in the group of patients on MD compared to the control group

Abbreviations: ASDAS-CRP, Ankylosing Spondylitis Disease Activity Score with C-reactive protein; axSpA, axial spondyloarthritis; BASDAI, Bath Ankylosing Spondylitis Disease Activity Index; BMI, body mass index; BSA, body surface area; CPDAI, composite psoriatic disease activity index; DAPSA, Disease Activity Index for PsA; ESR, Erythrocyte Sedimentation Rate; HAQ, Health Assessment Questionnaire; IL, interleukin; LEI, Leeds enthesitis index; MetS, metabolic syndrome; PASI, Psoriasis Area Severity Index; PREDIMED, PREvencion con DIeta MEDiterranea; PsA, psoriatic arthritis; PsO, psoriasis.

**Table 4:** Effects of MD on patients with CTDs.

Reference	Disease Type	Study Type	Number of Participants, Age, Gender Distribution	Diet Effects	Intervention	Outcomes
Barbhaiya et al., 2021 [138]	SLE	Prospective cohort study	Two cohorts of 79,568 and 93,554 women aged 30–55 years and 25–42 years, respectively, of whom 194 had SLE	Neutral–Positive	Administration of validated questionnaires on frequency of food intake at baseline and at follow-up with the calculation of 4 diet scores, including the Alternative Mediterranean Diet Score	No association between likelihood of SLE diagnosis and dietary habits, including MD. Decreased risk of SLE in subjects with the highest AHEI-2010 tertile of nut and legume intake
Pocovi-Gerardino et al., 2021 [30]	SLE	Cross-sectional study	280 patients aged 46.9 ± 12.8 years, 90.4% of whom were female	Positive	Assessment of MD adherence using the PREDIMED questionnaire and assessment of disease activity and damage accrual using SLEDAI-2K, SLICC/ACR and SDI	Significant association between higher PREDIMED scores and fewer CV risk factors. Significant association between higher PREDIMED scores and lower SLEDAI and SDI scores. Significantly lower SLEDAI and SDI scores among consumers of MD food components such as vegetables and fruit, fish and olive oil
Gavilán-Carrera et al., 2024 [141]	SLE	Cross-sectional study	76 women with SLE in mild disease activity aged 43.5 ± 13.8 years	Negative	Evaluation of adherence to the MD using the 11-item Mediterranean Diet Score and SLE disease activity using the SLEDAI score	Negative association between consumption of whole dairy products and glucorticoid intake and dosage. Positive correlation between red wine consumption and the likelihood of taking immunosuppressants
Vordenbäumen et al., 2023 [139]	SLE	Cross-sectional study	145 patients aged 44.3 ± 31.7 years, of whom 87.6% were female	Neutral	Assessment of MD adherence, energy expenditure for physical activity, depression, fatigue and SLE disease activity using the MEDAS, PAEE, CES-D, FSS and SLEDAI questionnaires	Healthy lifestyle recorded in 49 SLE patients, which correlated with a better physical quality of life, lower fatigue and depression and reduced titers of anti-dsDNA antibodies. Higher impact of physical activity on health compared to dietary pattern
DelOlmo-Romero et al., 2024 [140]	SLE	Cross-sectional study	293 patients aged 46.85 ± 12.9 years, of whom 90.4% women	Neutral	Assessment of participants on MetS by applying the National Cholesterol Education Program Adult Treatment Panel III criteria, SLE disease activity and damage accrual by SLEDAI-2K and SDI and MD adherence by a 14-item questionnaire	No significant association between MD adherence and disease activity or damage accrual. MetS recorded in 15% of SLE patients and significantly associated with SDI scores and complement C3 levels
Tsoi et al., 2024 [142]	SLE	Systematic review	More than 1000 participants from 102 studies, of which 15 on diet and nutrition	Positive for SLE-related CV risk	Search for evidence of the effectiveness of diet and healthy lifestyle on SLE disease activity in the Medline, Embase, Web of Science and Cinahl databases	Lower CV risk with the consumption of MD foods
Machowicz et al., 2020 [150]	pSS	Case–control study	82 patients (77 women, mean age ± SD 56 ± 14 years) and 51 sicca patients (47 women, mean age ± SD 57.4 ± 11 years)	Positive	Evaluation of nutritional behavior using a semi-quantitative MD score	Inverse correlation between the MD total score and the probability of pSS. Inverse correlation between frequency of fish consumption, intake of galactose, vitamin A/retinol equivalents and vitamin C and the likelihood of pSS.
Carubbi et al., 2021 [31]	pSS	Cross-sectional study	93 patients (95% female, mean age, SEM 61.8, 1.2 years)	Positive	Assessment of MD adherence with the PREDIMED and MEDLIFE questionnaires; assessment of disease activity with the ClinESSDAI	Good, moderate and low adherence to MD according to the PREDIMED questionnaire found in 31%, 61% and 8% of pSS participants, respectively. Inverse correlation between the PREDIMED score and the ClinESSDAI score.
Natalello et al., 2024 [32]	SSc	Cross-sectional study	387 patients (94.6% female, mean age ± SD 55.6 ± 13.9 years)	Positive	Assessment of MD adherence through the 14-MEDAS questionnaire, severity of gastrointestinal symptoms, depression and anxiety and work productivity	Negative correlation between the 14-MEDAS score and gastrointestinal symptoms, depression and anxiety scores, severity of Raynaud’s phenomenon and work productivity impairment

Abbreviations: AHEI-2010, Alternative Healthy Eating Index; CES-D, Depression Scale; ClinESSDAI, Clinical Eular Sjögren’s Syndrome Disease Activity Index; FSS, Fatigue Severity Scale; MD, Mediterranean diet; MEDAS, Mediterranean Diet Adherence Score; MEDLIFE, Mediterranean lifestyle index; MetS, Metabolic Syndrome; PAEE, physical activity energy expenditure; PREDIMED, PREvencion con DIeta MEDiterranea; pSS, primary Sjögren’s syndrome; SSc, systemic sclerosis; SD, standard deviation; SDI, SLICC/ACR Damage Index; SEM, standard error of the mean; SLE, systemic lupus erythematosus; SLEDAI-2K, Systemic Lupus Erythematosus Disease Activity Index 2000; 14-MEDAS, 14-item Mediterranean Diet Adherence Screener.
